# Inter-Individual Differences in Neurobehavioural Impairment following Sleep Restriction Are Associated with Circadian Rhythm Phase

**DOI:** 10.1371/journal.pone.0128273

**Published:** 2015-06-04

**Authors:** Tracey L. Sletten, Ahuva Y. Segal, Erin E. Flynn-Evans, Steven W. Lockley, Shantha M. W. Rajaratnam

**Affiliations:** 1 Sleep and Circadian Medicine Laboratory, School of Psychological Sciences, Monash University, Melbourne, Victoria, Australia; 2 Division of Sleep and Circadian Disorders, Departments of Medicine and Neurology, Brigham and Women's Hospital; Division of Sleep Medicine, Harvard Medical School, Boston, Massachusetts, United States of America; Centre for Chronobiology, SWITZERLAND

## Abstract

Although sleep restriction is associated with decrements in daytime alertness and neurobehavioural performance, there are considerable inter-individual differences in the degree of impairment. This study examined the effects of short-term sleep restriction on neurobehavioural performance and sleepiness, and the associations between individual differences in impairments and circadian rhythm phase. Healthy adults (n = 43; 22 M) aged 22.5 ± 3.1 (mean ± SD) years maintained a regular 8:16 h sleep:wake routine for at least three weeks prior to laboratory admission. Sleep opportunity was restricted to 5 hours time-in-bed at home the night before admission and 3 hours time-in-bed in the laboratory, aligned by wake time. Hourly saliva samples were collected from 5.5 h before until 5 h after the pre-laboratory scheduled bedtime to assess dim light melatonin onset (DLMO) as a marker of circadian phase. Participants completed a 10-min auditory Psychomotor Vigilance Task (PVT), the Karolinska Sleepiness Scale (KSS) and had slow eye movements (SEM) measured by electrooculography two hours after waking. We observed substantial inter-individual variability in neurobehavioural performance, particularly in the number of PVT lapses. Increased PVT lapses (r = -0.468, p < 0.01), greater sleepiness (r = 0.510, p < 0.0001), and more slow eye movements (r = 0.375, p = 0.022) were significantly associated with later DLMO, consistent with participants waking at an earlier circadian phase. When the difference between DLMO and sleep onset was less than 2 hours, individuals were significantly more likely to have at least three attentional lapses the following morning. This study demonstrates that the phase of an individual’s circadian system is an important variable in predicting the degree of neurobehavioural performance impairment in the hours after waking following sleep restriction, and confirms that other factors influencing performance decrements require further investigation.

## Introduction

Sleep restriction impairs waking alertness, mood and neurobehavioural performance, including sustained attention, cognitive speed, cognitive accuracy and reaction time [1–3], in a dose-dependent [1, 4] and cumulative manner [1, 5]. Systematic variability in the degree of sleepiness and cognitive impairment following sleep loss has been observed between individuals [2, 6–9], and may account for as much as 83% of the observed variance in neurobehavioral responses to sleep loss [10]. While some individuals appear to be relatively resilient to the effects of sleep loss, others are particularly susceptible to impairment [2, 8, 11]. Inter-individual differences in impairment to sleep loss increase with higher sleep pressure [9], and are consistently observed within subjects across multiple exposures and across multiple neurobehavioural domains including sustained attention, selective attention, serial reaction time and scales for global vigor [2, 7, 12, 13]. These studies indicate that there are systematic trait-like differences in response to sleep loss.

The underlying basis of individual vulnerability in response to sleep loss is not well understood. Studies have suggested that aging may reduce sensitivity to sleep loss-related cognitive impairment [14–16] and that gender may play a role in individual response [17]. Although there appears to be variation in the amount of sleep required by an individual to maintain performance [18, 19], including reports of higher sleep need in women than men [20–22], others [23] indicate that individual differences in performance impairment following sleep loss are not determined by basal sleep need.

Inter-individual variability in alertness and performance is also associated with the phase relationship between the circadian system and the sleep-wake cycle. Numerous investigations have revealed large variability between individuals in circadian phase and in the phase angle of entrainment between sleep and circadian phase, even under controlled conditions of sleep timing and light exposure [24–30]. The role of phase angle in determining the time course of alertness is illustrated in totally blind individuals without light perception who can have broad differences or changes in circadian phase while trying to maintain a 24-hour sleep pattern. Individuals with advanced circadian rhythms, who wake at a later phase of the circadian cycle, demonstrate an earlier peak in alertness and performance with a rapid decline across the day, while delayed individuals demonstrate poorer morning alertness [31]. Non-entrained blind patients alternate between these two patterns as their circadian phase runs in and out of phase with the 24-hour sleep pattern [31]. The circadian phase at which waking occurs also strongly influences diurnal preference; morning types tend to have a shorter circadian cycle [32] and, while their circadian phase is relatively early, sleep is not advanced in parallel, resulting in morning types initiating sleep and waking up relatively later in their circadian cycle [32–36]. Evening types tend to have a longer circadian period, and a delayed circadian phase, but initiate sleep and wake up relatively earlier in their circadian cycle. Consequently, morning types are more alert in the morning (as they wake at a later phase of their circadian cycle) and evening types are more alert in the evening [33].

The degree to which inter-individual variability in neurobehavioural responses to sleep restriction is associated with circadian phase angle is not known. We studied the effects of two nights of sleep restriction on neurobehavioural performance, and examined associations between the circadian phase of entrainment and neurobehavioural performance.

## Materials and Methods

### Participants

Forty three healthy young adults (22 male, 21 female) aged 22.5 ± 3.1 (mean ± SD) years participated in this dual-site study conducted at Monash University, Melbourne (n = 31, 16 male) and the Brigham and Women’s Hospital (BWH), Boston (n = 12, 6 male). Volunteers were recruited via poster and web advertisements. Participants were healthy, as determined by physical examination, blood biochemistry and haematology, and electrocardiography. They reported normal sleep, were non-smokers, had body mass index between 18.5 and 30.5 kg/m^2^, and were not currently taking prescription medication other than the contraceptive pill. Individuals were excluded if they consumed high amounts of caffeine (> 300 mg/day) or alcohol (> 14 standard drinks/week) or reported taking illicit drugs in the previous 12 months. Participants reported that they were not working regular night shifts and had not travelled across more than two time zones in the prior three months. All reported a habitual bedtime between 9:00 pm and 2:00 am, habitual wake time between 5:00 am and 10:00 am, and habitual sleep duration of 7–9.5 hours. Diurnal preference was measured using the Morningness-Eveningness Questionnaire (MEQ) [37] and individuals with extreme scores below 30 or above 70 were excluded. The protocol was approved by the Monash University Human Research Ethics Committee, The Alfred Human Research Ethics Committee and the Partners Human Research Committee, and written informed consent was obtained from each participant prior to study.

### Pre-laboratory assessment

For at least 9 days prior to the laboratory session participants maintained a fixed, self-selected sleep-wake schedule (8 hours time-in-bed [TIB]) confirmed by time-stamped call-ins (telephone messages) recorded at bedtime and wake time, sleep diaries and wrist actigraphy (Respironics Inc, Bend, OR, USA). Participants refrained from taking prescription and non-prescription drugs including alcohol and nicotine throughout the study and abstain from caffeine for four days prior to the laboratory session.

On the night at home prior to the laboratory visit participants were instructed to delay their bedtime by 3 hours and wake at their regular time, thereby restricting TIB to 5 hours. During the extended wake, participants were instructed to remain in dim lighting (i.e., no overhead lighting), verified by an ambulatory light logger worn around the neck (HOBO, OneTemp Pty Ltd, Melbourne) in the Melbourne participants (70%, n = 30). Median light level recorded during extended wake was 7.2 ± 27.7 lux.

### Laboratory protocol

**Fig 1:**
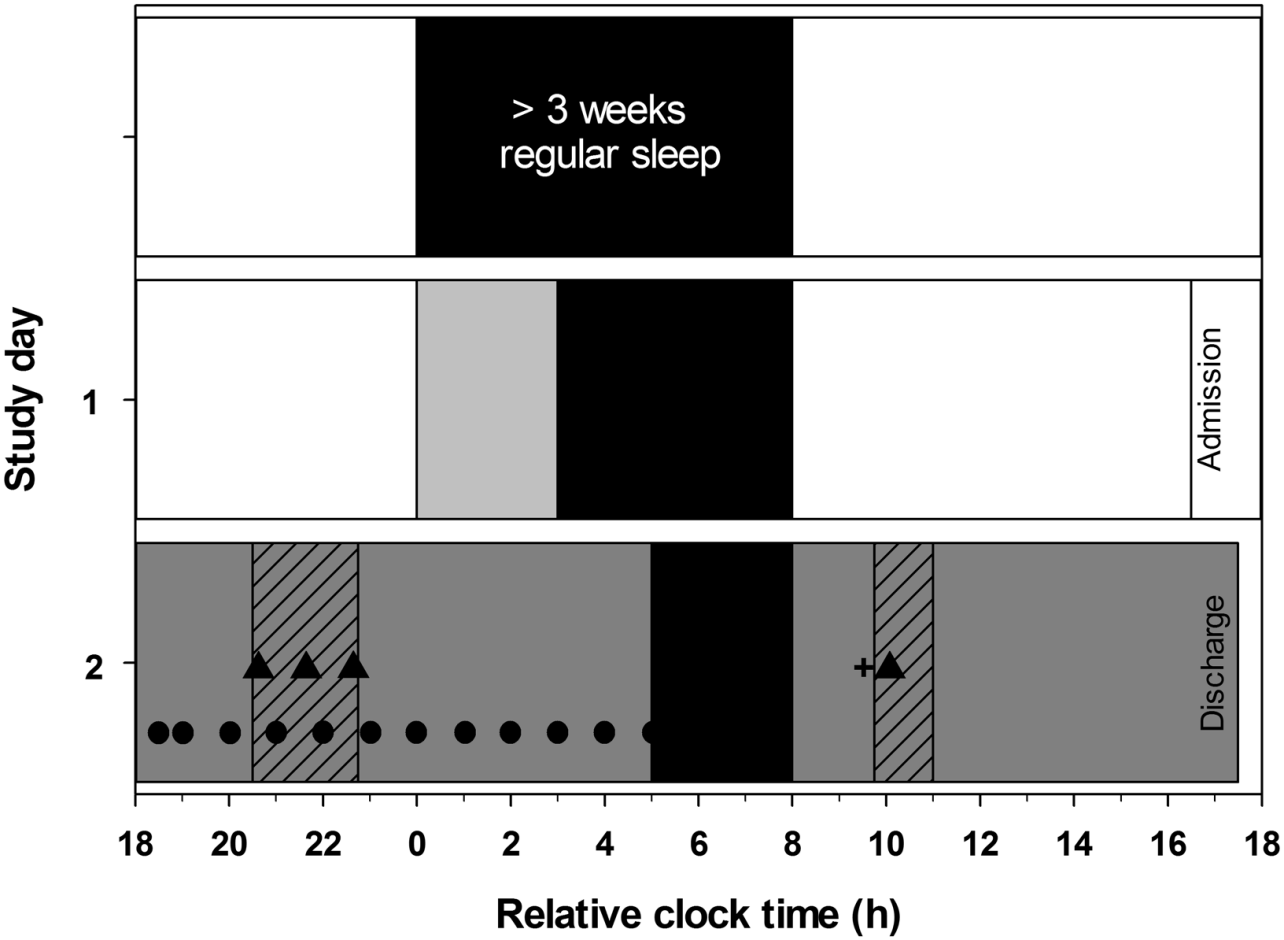
Study protocol to assess sleepiness following sleep restriction. The protocol is plotted for a participant maintaining a 00:00–08:00 h sleep schedule (black bars). Following at least 3 weeks of an 8-hour sleep schedule at home, participants restricted their sleep to 5 hours on the last night at home, remaining in dim light for the 3 hours of extended wake (grey bar on study day 1). Participants attended the sleep laboratory on the following evening. Two hours after session start ambient light levels were reduced to < 2 lux (dark grey bar on study day 2). Saliva samples (●) were collected every 30 to 60 minutes for assessment of circadian phase. Sleep was further restricted to three hours duration in the laboratory (0 lux). POMS-Bi (+) was completed 1.75 hours after waking. Two hours after waking participants underwent constant posture (grey diagonal striped bar) and completed a performance battery (▲) including the Karolinska Sleepiness Scale, auditory psychomotor vigilance test, and Karolinksa Drowsiness Test. The test battery was practiced up to three times during constant posture on study night 1.

Participants were studied in a time-free environment for approximately 26 hours (Fig 1). Participants arrived 6.5 hours before their routine bedtime and immediately provided a urine sample to be tested for drugs of abuse. Polysomnographic electrodes were attached to the scalp and face throughout the laboratory session according to the International 10–20 System with linked mastoid references (M1 and M2); frontal, central, parietal, occipital. Only data from two electrooculogram (EOG) derivations (LOC-M2, ROC-M1) are presented here. Polysomnographic recordings were made continuously during the constant posture testing periods and during sleep episodes using an ambulatory digital polysomnographic recorder (Siesta 802, Compumedics Limited, Victoria, Australia, sampling rate 256 Hz; Vitaport-3 digital recorder, TEMEC Instruments B.V., Kerkrade, The Netherlands, sampling rate 128 Hz). Electrode impedances were maintained at < 10 K ohms.

Saliva samples were collected in dim light 5.5 and 5 h before scheduled pre-laboratory bedtime, then hourly until 5 h after scheduled bedtime and immediately stored at −20°C [24]. Participants were seated for ≥ 20 min before each sample, and did not consume any food or beverages within 10 min of the sample. Participants were required to remain awake throughout the saliva collection phase. Sleep was permitted immediately after the last sample was collected, 5 h after scheduled bedtime.

From 3 h after arrival in the laboratory participants completed three trials of a neurobehavioral test battery, each separated by 1 h. Practice assessments were completed under constant posture conditions in which participants were supervised while they remained awake in a seated posture. Between completion of practice tests and the sleep opportunity participants were free to read or play board games. Participants were given a 3-hour sleep opportunity, by delaying their scheduled bedtime by 5 hours and waking up at their scheduled waketime. One hour after waking, participants began a constant posture protocol in which they remained seated at the computer and regularly completed a battery of neurobehavioural tests beginning two hours after waketime. Between tests participants were permitted to read or play board games.

Ambient light levels were maintained at < 3 lux when measured vertically at a height of 183 cm for all wake periods (Melbourne: 36 W PL-L 840 fluorescent lamps, 4000 K, Philips Lighting, Eindhoven, The Netherlands; Lee neutral density filters, Lightmoves Pty Ltd, Melbourne, Australia; Boston: 4100K fluorescent lamps F96T12/41U/HO/EW, 95W; F32T8/ADV841/A, 32W; F25T8/TL841, 25W; Philips Lighting, The Netherlands; Lextran 9030 with prismatic lens, GE Plastics, MA). Ambient light was reduced to 0 lux during the sleep opportunity. On the first night in the laboratory, a meal was provided 5.25 h after arrival and a snack provided 8.5 h after arrival. On the following day, a standardised meal was provided 0.5 h after waking.

### Neurobehavioural performance assessments

The neurobehavioural test battery was administered on a computer using the software E-Prime 2.0 (Psychology Software Tools Inc., Pittsburgh, PA, USA), and included the Karolinska Sleepiness Scale, an auditory 10-minute Psychomotor Vigilance Task, the Karolinska Drowsiness Test for three minutes with eyes open, and the Profile of Mood States.

*Karolinska Sleepiness Scale (KSS)* [38] was used to self-report level of sleepiness in the preceding 10 minutes with a scale from 1 = ‘very alert’ to 9 = ‘very sleepy, fighting sleep’.

*Psychomotor Vigilance Task (PVT)* [11] was used to assess sustained attention, with participants responding to an auditory stimulus as quickly as possible by pressing the spacebar on a standard computer keyboard using their dominant hand. The inter-stimulus interval varied between 2 and 10 seconds. The number of lapses in attention, defined as reaction times longer than 500 msec, was calculated for each 10-minute task.

*Karolinska Drowsiness Test (KDT)* [38] was used to assess slow eye movements (SEMs). Participants focussed their sight for 3 minutes on a 3 cm dot on the computer screen approximately 60 cm in front of the face. EOG record was visually scored for the proportion of 30-second epochs containing at least one SEM.

*Profile of Mood States Bi-Polar Form (POMS-Bi)* [39] was administered on one occasion, 1.75 hours after waking, to assess subjective mood (72 adjectives; 0 = much unlike this, 3 = much like this). The presented adjectives are categorised into six mood states or sub-scales; Agreeable-Hostile, Clearheaded-Confused, Composed-Anxious, Confident-Unsure, Elated-Depressed and Energetic-Tired, with higher values indicating more positive mood state.

### Data analysis

Saliva samples were analysed for melatonin concentration via radioimmunoassay [40] with a limit of detection of 1 pg/ml. Dim light melatonin onset (DLMO) was determined as the time that melatonin concentrations crossed and remained above a threshold of 10 pM (or 2.3 pg/ml), as described previously [41]. The phase angle between DLMO and pre-laboratory sleep time was calculated by subtracting DLMO time from sleep onset time. Sleep onset time was calculated as the average time of sleep onset for the 9 nights prior to the laboratory visit, as determined from sleep diaries, daily call-ins and actigraphy. Subjective report of bedtime was used to identify the start of sleep episodes for actigraphic analysis. Sleep onset was established by identifying at least 10 consecutive one-minute epochs in which no more than 1 epoch contained measured activity. The first epoch of this window was scored as sleep onset. The final sleep at home on the night prior to the laboratory visit was excluded from the calculation of sleep onset because participants were required to delay their bedtime.

The following outcome measures were assessed two hours after waking: KSS score, number of lapses on the PVT, and percentage of EOG epochs containing SEMs during the KDT. To examine the relationship between the number of PVT lapses and circadian phase, participants were ranked according to the number of lapses and categorised as recording no lapses (n = 16) or at least 4 lapses (n = 16). The threshold of 4 lapses was adopted based on the mean number of lapses recorded by all participants (4.30 ± 5.61 lapses, see results). Differences were examined by independent samples t-test. A Chi-square test for independence with Yates Continuity Correction was applied to examine the risk of recording lapses in attention on the PVT when phase angle was shorter than the average of two hours.

Pearson Product Moment correlations were used to examine associations between circadian phase (i.e., DLMO and phase angle of entrainment) and measures of sleepiness (i.e., KSS score and SEMs) two hours after waking, and circadian phase and the number of PVT lapses. Chi-square test was used to examine whether phase angle difference (< 2 h average reported in the literature) [24–27] was associated with increased number of lapses (≥ 3 lapses) on the PVT. Pearson Product Moment correlations were used to examine associations between circadian phase (i.e., DLMO and phase angle of entrainment) and subjective mood (Bi-POMS).

To examine the proportion of variance in PVT lapses that was explained by circadian phase relative to other variables, a linear regression with backward variable selection with an inclusion threshold of p = 0.05 was performed. Factors incorporated into the model were phase angle of entrainment, age, gender, BMI, and morningness-eveningness score.

## Results

On average, individuals recorded 4.30 ± 5.61 lapses per 10-minute PVT, ranging from 0 to 21 lapses (n = 43). Mean subjective sleepiness (KSS score) was 6.42 ± 1.76 (range = 3 to 9, n = 43). The proportion of 30-second EOG epochs containing SEMs during the 3-minute KDT ranged from 0% to 83% with an average of 24.86 ± 31.16% (n = 37).

Table 1 shows comparisons between individuals recording no PVT lapses and individuals who recorded four or more lapses (n = 32). These two groups did not significantly differ in age, BMI or MEQ score (see Table 1). Although the two groups did not differ in sleep onset time (t(29.4) = -1.31, p > 0.05), individuals in the no lapse group had significantly earlier circadian phase as determined by the clock time of DLMO (t(24.3) = -3.52, p < 0.01). Consequently, sleep onset times for the no lapse group were significantly later relative to DLMO (i.e., significantly larger phase angle difference between DLMO time and sleep time) (t(28.2) = 3.44, p < 0.01).

**Table 1:** Participant characteristics shown for the entire sample (n = 43) and separately for individuals recording no lapses on the PVT (n = 16) compared to individuals with a high number of lapses (4+, n = 16) two hours after waking.

	Range	mean ± SD	No lapses	4+ lapses	p*
			(mean ± SD)	(mean ± SD)	
Gender	22 M, 21 F		5 M, 11 F	10 M, 6 F	0.077
Age (years)	18.4–31.0	22.53 ± 3.07	23.06 ± 3.67	22.40 ± 2.79	NS
Body Mass Index (kg/m2)	18.7–30.5	22.34 ± 2.83	22.31 ± 2.66	21.93 ± 3.21	NS
Horne-Ostberg Questionnaire	38–70	53.55 ± 7.45	54.97 ± 6.81	51.53 ± 6.73	NS
Sleep onset time (h)	21:51–24:58	23:48 ± 00:55	23:34 ± 0:51	23:59 ± 0:59	NS
Dim Light Melatonin Onset (h)	19:06–01:00	21:38 ± 01:23	20:52 ± 0:55	22:27 ± 1:33	0.001
Phase angle difference (dec h)	-0.31–4.14	2.16 ± 1.08	2.70 ± 0.83	1.54 ± 1.07	0.002

* Characteristics between groups were compared using Student’s t-test. Gender was compared using Chi-Squared test.

**Fig 2:**
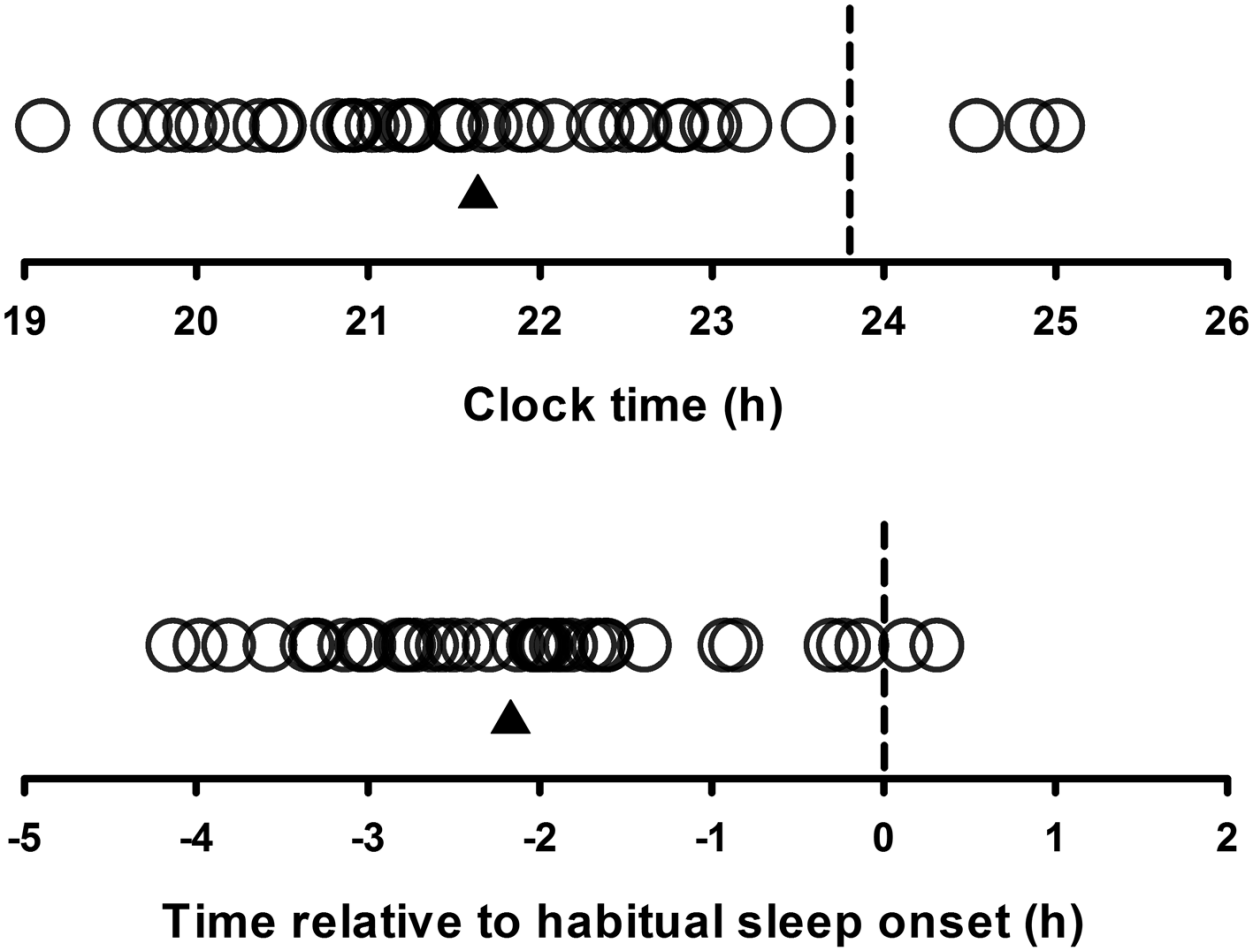
Timing of melatonin onset (circles) for each participant (n = 43) relative to clock time (upper panel) and relative to sleep onset (phase angle of entrainment; lower panel). Dashed line represents mean time of sleep onset and the triangle represents the mean melatonin onset time for all participants. A subset of these data (n = 28) has been published previously [24].

**Fig 3:**
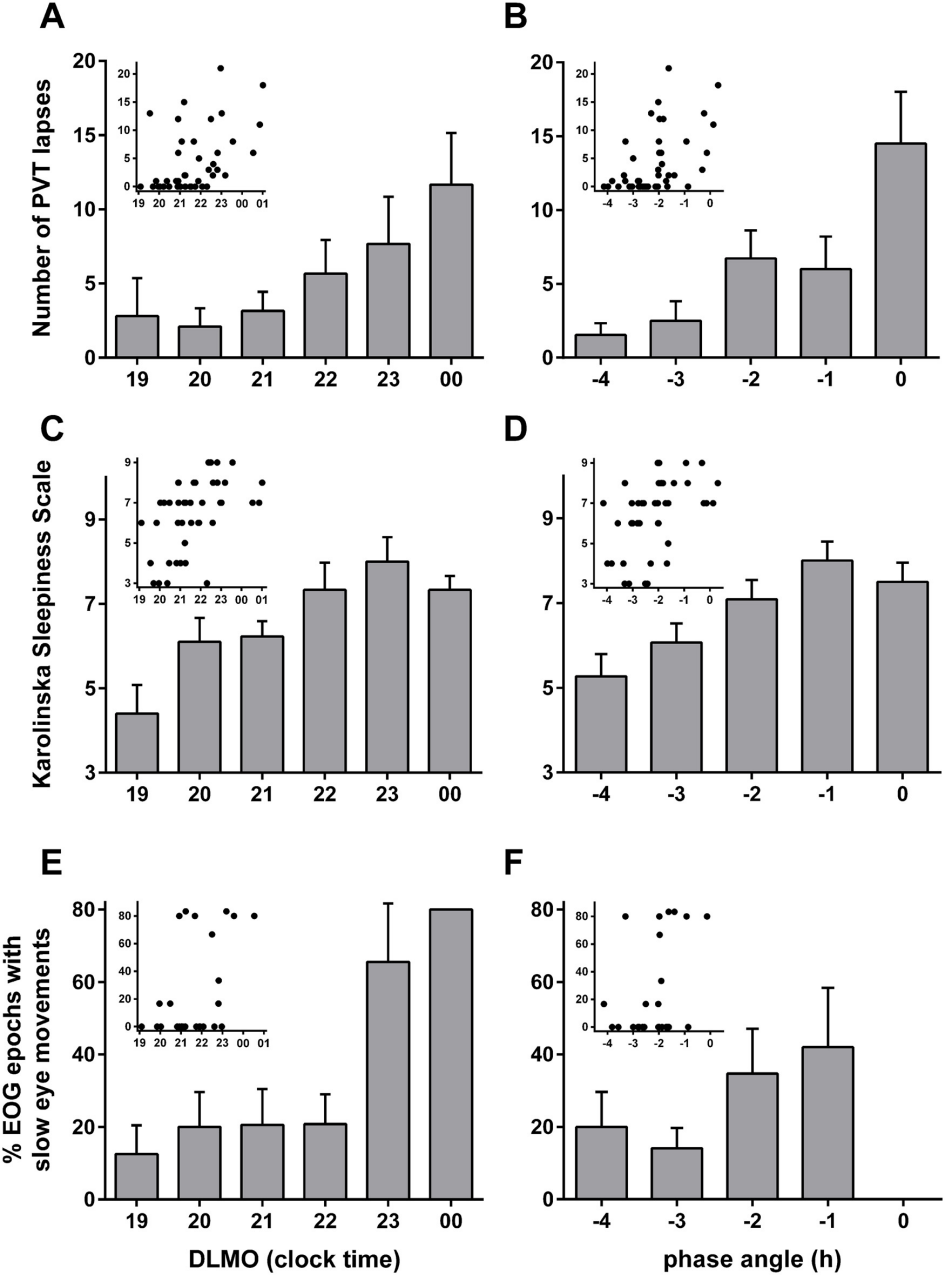
Sleepiness measures two hours after waking following two nights of sleep restriction, as indicated by the number of PVT lapses (A & B; n = 43), Karolinska Sleepiness Scale (C & D; n = 43), and the proportion of EOG epochs containing slow eye movements (E & F; n = 37). Data are plotted relative to circadian phase represented by (a) the clock hour of an individuals’ DLMO (left panels; A, C, E) and (b) phase angle difference between dim light melatonin onset (DLMO) and mean sleep onset on the 9 nights prior to the laboratory visit (right panels; B, D, F), (e.g., -2 represents someone with a DLMO occurring 2 hours before sleep onset time). Main bar plots represent data categorised into hourly bins (mean ± SEM) to illustrate the relationship between circadian phase and sleepiness. Inserts present the raw data scatterplots.

Fig 2 illustrates the wide degree of variability in melatonin onset time between participants. Significant associations were observed between PVT lapses and DLMO (n = 43, r = -0.468, p < 0.01), and between PVT lapses and phase angle difference (n = 43, r = -0.510, p < 0.0001), with a later DLMO time and shorter phase angle (i.e., less difference between sleep time and DLMO time) associated with more lapses (Fig 3).

A Chi-square test for independence (with Yates Continuity Correction) indicated that when the phase angle difference between sleep time and DLMO time was less than 2 hours, individuals were significantly more likely to have three or more lapses on the PVT; χ^2^(1, n = 43) = 9.68, p <0.005, phi = 0.52. Twenty percent of individuals with a phase angle shorter than 2 hours had three or more lapses on the PVT compared to 72% of individuals with a phase angle of 2 hours or longer. Regression analysis of variables associated with the number of PVT lapses resulted in stepwise exclusion of age, gender, BMI, and morningness-eveningness. The final model included only phase angle of entrainment between DLMO and sleep onset (*F*_1,42_ = 14.41, R square = 0.260, p < 0.0001). In post-hoc analysis, because the distribution was found to violate assumption of normality (W = 0.782, p < 0.001), we repeated the analysis using a Poisson model with phase angle difference to confirm the association with the number of PVT lapses. We found a significant relationship between phase angle and PVT lapses (Wald χ^2^ = 75.93, Exp(B) = 0.575, p < 0.001).

Consistent with the relationships between DLMO time and PVT lapses, later DLMO time was also associated with higher subjective sleepiness (KSS) ratings (n = 43, r = 0.510, p < 0.0001; Fig 3) and higher number of EOG epochs containing slow eye movements (n = 37, r = 0.375, p = 0.022; Fig 3) 2 hours after waking. Phase angle was negatively correlated with KSS (n = 43, r = -0.483, p = 0.001) and the relationship between phase angle and number of EOG epochs containing slow eye movements approached significance (n = 37, r = -0.311, p = 0.061).

**Fig 4:**
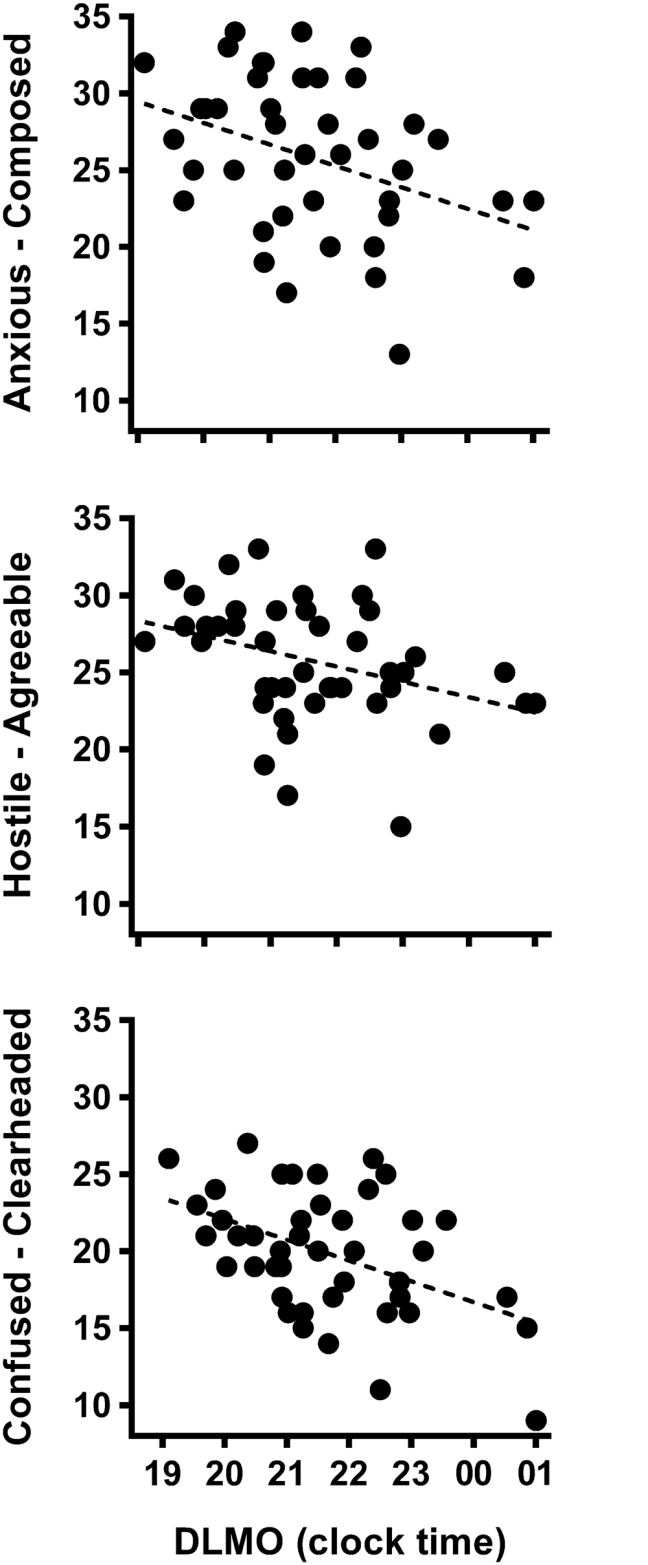
Relationships between dim light melatonin onset and mood states composed-anxious (r = -0.366), agreeable-hostile (r = -0.347) and clearheaded-confused (r = -0.459). All relationships were significant (p < 0.05). Higher values represent more positive mood.

Later DLMO time was associated with poorer mood ratings approximately 2 h after waking: composed (n = 43, r = -0.366, p = 0.016), agreeable (n = 43, r = -0.347, p = 0.022) and clear-headed (n = 43, r = -0.459, p = 0.002) (Fig 4). Relationships between later DLMO and lower levels of confidence (n = 43, r = -0.270, p = 0.084) and energy (n = 43, r = 0.268, p = 0.083), and relationships between longer phase angle and higher agreeable (r = 0.27, p = 0.086) and energetic (r = 0.26, p = 0.092) mood states were not statistically significant.

## Discussion

The study demonstrates significant inter-individual variability in sleepiness, performance and mood, particularly in the number of attentional (PVT) lapses, when assessed two hours after waking following two nights of sleep restriction. Later circadian phase was associated with increased attentional lapses, higher sleepiness ratings, more slow eye movements and poorer mood outcomes. Shorter phase angle difference between DLMO and sleep (i.e., sleeping and waking at an earlier phase of the circadian cycle) was associated with poorer performance and higher sleepiness two hours after waking.

The degree of short-term sleep restriction employed in the current study was sufficient to cause impairments in alertness. The mean number of PVT lapses (~4 lapses) recorded two hours after waking is similar to the mean lapses observed at three points across the day (1000, 1600, 2200 h) in a previous study after seven nights in which time-in-bed was restricted by 33% of habitual sleep duration (~5 hours) [5]. This finding is consistent with a previous study showing that neurobehavioural deficits after a more rapid accumulation of sleep loss are greater than when the same magnitude of sleep loss is accumulated over a longer interval [42]. In our study, participants recorded fewer PVT lapses than the daily average observed after two nights of 3 hours time-in-bed [1] but more than that observed following two nights of exposure to 4 hours time-in-bed [4], or 5 hours time in bed [1], when controlled for time of day. It should be noted, however, that the current study administered an auditory version of the PVT which has been associated with less frequent lapses [43] than the visual version of the task (1.37 ± 0.33 lapses on the auditory PVT vs 8.05 ± 11.37 lapses on the visual PVT on a baseline day). In our study, the mean level of performance impairment on the PVT may be greater than that observed in the previous studies administering the visual PVT.

Substantial inter-individual variability in sleepiness was observed in the number of lapses in attention during the PVT. While a substantial proportion of individuals did not have any lapses in attention (37%), other individuals recorded as many as 18 and 21 lapses during the 10-minute PVT. The finding of systematic individual differences in the number of lapses on the PVT supports previous research showing a range of more than 20 lapses [2, 10]. The individual differences in lapses (as determined by the between-subject standard deviation for the random intercept) is comparable to the magnitude of impairment following 3.2 days of sleep restricted to 4 hours [44].

Consistent with previous studies [25–27] and with a preliminary report from the present study [24], DLMO occurred on average ~2 hours prior to bedtime. The results also revealed, however, a large range of variability in the time of DLMO and its phase relationship to sleep onset time, confirming prior findings of inter-individual differences in phase and phase angle of entrainment [24–30, 45]. Differences in phase angle may result from variability in core properties of the circadian clock [25, 46], such as the length of the intrinsic period [32, 47, 48] or sensitivity of the phase-resetting effects of light [49–51].

Neurobehavioural performance impairment following sleep loss was associated with individual circadian phase, assessed as the time of DLMO. Higher levels of sleepiness according to the KSS and EOG measurements as well as increased impairment on the PVT two hours after waking were significantly associated with later timing of melatonin onset. Later melatonin rhythm phase was also associated with poorer mood in the domains of being composed, agreeable and clearheaded. The results indicate that endogenous circadian phase and the resultant phase angle may be an important predictor of the degree of daytime sleepiness and impairments in performance and mood due to sleep restriction. These findings are consistent with those of Van Dongen et al. [8], who report that performance on a serial addition/subtraction task following sleep restriction is significantly associated with core body temperature rhythm phase. This previous study, however, failed to demonstrate this association in other neurobehavioural performance measures including the PVT, leading the authors to conclude that circadian phase was not a reliable correlate of differential vulnerability to neurobehavioural impairment from sleep loss in their study. The findings of the present study challenge this view.

As a result of the inter-individual differences in circadian phase, coupled with societal restrictions on sleep such that people may not be able to choose to sleep at an optimal circadian phase, the phase angle of entrainment varies between individuals. A more delayed phase was associated with increased impairment due to testing occurring closer to the circadian trough, earlier in the circadian cycle. In addition, these individuals may also experience impaired sleep duration and efficiency in the early part of their sleep attempt due to attempting to sleep during their wake maintenance zone [52–54], as a result of their sleep timing being misaligned from the circadian clock. These individuals may experience symptoms of insomnia, thereby augmenting the impairment in waking performance due to poor sleep. Furthermore, circadian modulation of sleep inertia has also been reported, with the worst cognitive performance impairment when waking during the biological night [55]. This observation suggests that the degree of impairment due to sleep inertia upon waking is likely to be augmented in individuals with delayed circadian phase. In this case [55], circadian regulation of sleep inertia was examined 20 minutes after waking. The current study, however, assessed sleepiness two hours after waking to minimise the effects of sleep inertia. Preliminary analysis of data collected at 1 hour and 2 hours after waking in all participants showed that the number of PVT lapses was higher 2 hours after waking (M = 4.30, SD = 5.61) compared to 1 hour after waking (M = 2.72, SD = 4.17, p = 0.015), indicating the sleep inertia was not influencing alertness levels at the time of assessment.

The phase angle between sleep and circadian phase is one of two major determinants of diurnal preference [34, 56], in addition to changes in the rate of homeostatic accumulation of sleepiness [57]. Diurnal preference has been shown to influence cognitive performance during extended wakefulness [19, 58] and morning types tend to be more alert in the morning as they wake at a later phase of their circadian cycle [33]. The current results, however, demonstrated that morningness-eveningness was not significantly related to performance two hours after waking following sleep restriction, suggesting that diurnal preference is not a reliable marker of circadian phase. It should be noted, however, that individuals who were extremes in morningness-eveningness were excluded from study participation, thereby reducing the range of diurnal preferences in the sample.

**Fig 5:**
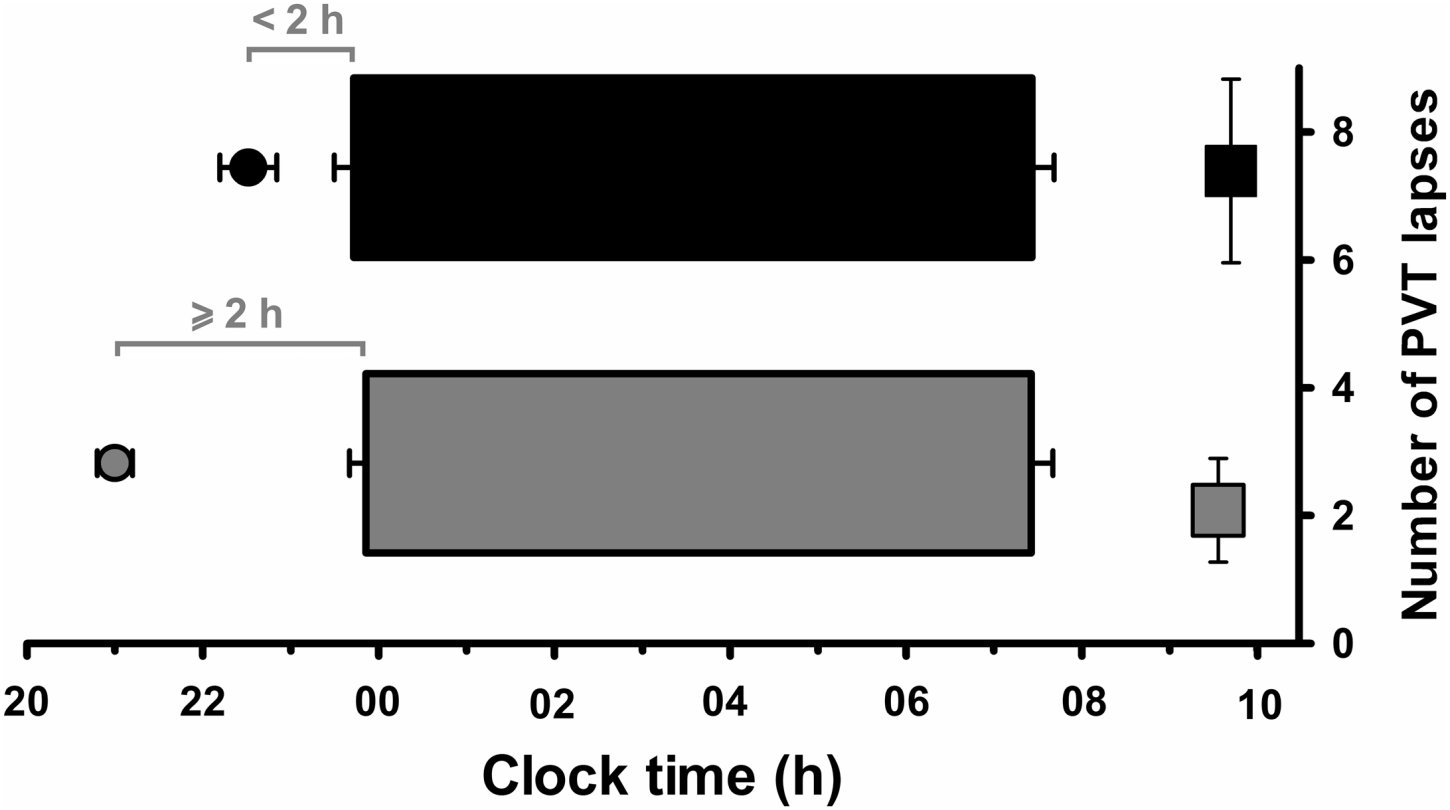
Schematic representation of the relationship between circadian phase and morning performance for individuals with a phase angle < 2 h (black) and individuals with a phase angle ≥ 2 h (grey). Phase angle of entrainment represents the time between dim light melatonin onset (DLMO) and mean sleep onset on the 9 nights prior to the laboratory visit. Circles illustrate the timing of DLMO; horizontal bars represent sleep times; squares represent the number of PVT lapses two hours after waking.

The current results indicate that circadian phase angle predicts poor performance and mood outcomes following sleep restriction. Specifically, our results suggest that individuals with a smaller phase angle of entrainment, those with a relatively more delayed circadian phase, are more vulnerable to the negative impact of sleep restriction and display increased sleepiness upon waking. Conversely, individuals with a larger phase angle, which indicates a longer time interval between melatonin onset and sleep onset time (sleeping and waking later in their circadian cycle), may not have the opportunity to express greater morning sleepiness following sleep restriction (Fig 5). These findings are consistent with the increased sleepiness and poorer mood outcomes observed in totally blind individuals who demonstrate an abnormally delayed rhythm, particularly in the hours following waking [59]. Together, this research indicates that studies of inter-individual differences in temporal performance due to sleep loss should also measure and account for circadian phase given the contribution of phase angle to performance at different time points.

The findings revealed a trend for gender to be significantly different between individuals who recorded a high number of PVT lapses compared to those not having any lapses, with more males performing poorly. Increased performance impairment in men compared to women following acute sleep deprivation has been previously reported for vigilance [60], verbal and visuo-constructive tasks [61], although there was no baseline assessment of performance in the latter study so differences may not directly reflect a differential response to sleep loss. Men and women also display differences in phase angle of entrainment, with the timing of circadian phase occurring later relative to sleep time in men compared to women [62]. The differences in this study may be associated with gender differences in circadian rhythm phase. The possibility that gender interacts with circadian phase to influence performance outcomes following sleep loss should therefore be examined further in future studies.

While our findings demonstrate a significant relationship between the phase of an individual’s circadian system and the degree of neurobehavioural performance impairment in the hours after waking following sleep restriction, there is a large proportion of the variance in PVT lapses that was not accounted for by circadian phase alone. Although we examined some of the variables that may contribute to inter-individual differences in neurobehavioural performance (i.e. age, gender, BMI), we did not account for all possible candidates. An underlying genetic basis to individual vulnerability to sleep loss has been proposed. The PER3 clock gene is associated with diurnal preference, with the variable number tandem repeat polymorphism 5-repeat allele (PER3^5/5^) associated with a greater preference for morningness [63], increased physiological sleepiness [64] and increased decrements in waking performance [64, 65] following sleep deprivation, compared to homozygosity for the shorter allele, PER3^4/4^. It is unclear, however, whether the increased vulnerability to sleepiness that occurs during total sleep deprivation in individuals with the PER3^5^ genotype is also observed following sleep restriction [66, 67].

This study is limited by the number of timepoints during which neurobehavioural performance was assessed after sleep restriction. Specifically, we limited our assessment of performance to up to two hours after waking. This study is part of a larger protocol examining the alerting effects of light exposure following sleep restriction. As such, assessments of sleepiness later in the day were confounded by light exposure and could not be examined. This restriction in the time of assessment to the morning may have augmented the influence of phase on sleepiness and performance after sleep restriction if some individuals were still on the descending limb of the circadian rhythm of sleep propensity. Repeated assessments beyond two hours after waking are required to determine whether the individuals with advanced and delayed circadian phase demonstrate differences in the time course of performance across the day as has been revealed previously for mood, alertness and performance [31]. Future studies should consider extending the duration of sleep restriction to examine the role of circadian phase on the response to a more cumulative degree of sleep loss as often occurs in modern working society.

Short-term sleep restriction, as assessed in this study is a common experience for many people [68]. Human error due to increased sleepiness as a result of sleep restriction can have significant consequences in terms of workplace accidents [69]. Consequently, in a work setting it may be important to consider the differences in vulnerability and implement targeted countermeasures to reduce the risk of human error. Our findings demonstrate that individuals with shorter phase angle difference between DLMO and sleep times are more vulnerable to neurobehavioural impairment in the morning hours after sleep restriction and therefore we would predict that those with a delayed phase are more likely to be at an increased accident risk on the drive to day work or at the start of the shift, or at the end of the shift or driving home after a night shift. Indeed, other populations experiencing delayed circadian phase, such as adolescents who demonstrate more evening tendency and wake at an earlier circadian phase [70], appear to be more vulnerable to motor vehicle crashes in the morning [71]. Individuals with delayed phase may also be more susceptible to poor mood associated with circadian misalignment [49, 72]. Circadian rhythm phenotyping may therefore provide valuable information in the management of the risk of daytime sleepiness, and may inform targeted intervention strategies such as individually-determined or more flexible working times, or appropriately timed circadian regulators such as exogenous melatonin or light.
